# Circulating interleukin-1β promotes endoplasmic reticulum stress-induced myocytes apoptosis in diabetic cardiomyopathy via interleukin-1 receptor-associated kinase-2

**DOI:** 10.1186/s12933-015-0288-y

**Published:** 2015-09-23

**Authors:** Zhongwei Liu, Na Zhao, Huolan Zhu, Shunming Zhu, Shuo Pan, Jing Xu, Xuejun Zhang, Yong Zhang, Junkui Wang

**Affiliations:** Department of Cardiology, Shaanxi Provincial People’s Hospital, No.257, Western Friendship Rd, Xi’an, People’s Republic of China

**Keywords:** Diabetic cardiomyopathy, Endoplasmic reticulum stress, Apoptosis, IL-1, IRAK-2

## Abstract

**Aim:**

IL-1β was considered as an important inflammatory cytokine in diabetic cardiovascular complications. DCM is one of the major manifestations of diabetic cardiovascular complications whose specific mechanisms are still unclear. In this study, we investigated the role of IL-1β in myocytes apoptosis in DCM.

**Methods:**

In the in vitro study, high- glucose medium and/or IL-1β were used to incubate the isolated primary myocytes. siRNA was used to knockdown the *irak2* gene expression. Apoptosis was evaluated by Hoechst and TUNEL staining. In the in vivo study, DCM in rats was induced by STZ injection and confirmed by cardiac hemodynamic determinations. The IL-1 receptor antagonist, IL-1Ra was also used to treat DCM rats. Myocardial apoptosis was assessed by TUNEL assay. In both in vitro and in vivo studies, expression levels of GRP-78, IRAK-2 and CHOP were analyzed by Western Blotting. ELISA was employed to exam the IL-1β content in serum and cell supernatants.

**Results:**

Myocytes were not identified as the source of IL-1β secretion under high- glucose incubation. High glucose incubation and/or IL-1β incubation elevated ER- stress mediated myocytes apoptosis which was attenuated by *irak2* silencing. Dramatically increased circulating and myocardial IL-1β levels were found in DCM rats which stimulated activation of ER stress and lead to elevated myocytes apoptosis. The administration of IL-1Ra, however, attenuated IRAK2/CHOP induced apoptosis without affecting fasting blood glucose concentration.

**Conclusions:**

Elevated circulating IL-1β contributed to promote ER stress- induced myocytes apoptosis by affecting IRAK-2/CHOP pathway in DCM.

## Background

In worldwide, the rapidly growing morbidity and mortality of both type-1 and type-2 diabetes are growing fast [1, 2]. Among the complications of diabetes, cardiovascular complications were considered contributing largely to the mortality of diabetes because of diabetic cardiomyopathy (DCM), myocardial infarction and sudden cardiac death [3]. As one of the most important manifestations of diabetic cardiovascular complications, DCM is characterized by impaired cardiac diastolic and systolic functions [4]. Though the mechanisms of DCM are still unclear, our previous studies proved that contractile units lost which was caused by myocardial apoptosis took responsibility for cardiac dysfunction in DCM [5].

Accumulating studies suggested that diabetes was one of the inflammatory diseases, which was evidenced by the elevated levels of cytokines and chemokines in patients with diabetes [6]. Further studies reported inflammatory cytokines such as interleukin-1β (IL-1β) were considered predictive of prognosis of type- 2 diabetes [7]. On one hand, excessive IL-1β was found produced by T cells and pancreatic β-cells under circumstance of hyperglycemia [8]; on the other hand, the elevated IL-1β induced apoptosis of β-cells to further impair insulin secretion [6]. Therefore, IL-1β plays an important role in the pathogenesis of diabetes. Other studies indicated that cardiomyocyte apoptosis was induced by extracellular IL-1β [9]. Results from these studies aroused our interests to implement further investigation on the association between IL-1β and cardiovascular complications of diabetes, DCM especially.

As one of the critical organelles, endoplasmic reticulum (ER) carries out fundamental biological functions including protein folding, protein modification, lipid synthesis and calcium signaling [10]. Under stressful conditions, as the unfolded or misfolded protein accumulating in ER lumen, ER stress is triggered to initiate pathological changes. Our previous study confirmed that ER stress was one of the critical triggers of cardiomyocyte apoptosis in DCM [11], which was responsible for the myocardial cell loss in DCM [11, 12]. As a result of signaling activation in ER stress, pro- apoptotic factors such as C/EBP homologous protein (CHOP) would lead to programmed cell death [13]. The signaling network activating CHOP in ER stress is quite complex and still not completely clear. A recent study reported that expression of interleukin-1 receptor-associated kinase-2 (IRAK-2) was increased during ER stress and further promoted CHOP- induced cell apoptosis [14]. Other previous investigation established the notion that IRAK2 was the down-stream effector of IL-1 signaling which was one of the triggers of ER stress [15]. These results provided us more clues to study the inflammatory signaling in ER stress- mediated cell apoptosis.

After binding with IL-1β, the IL-1 receptor (IL-1R) is activated and then associates an accessory protein named AcP [16]. The complex further recruits the adapter protein myeloid differentiation protein 88 (MyD88) to initiate IRAK-2 pathways [17]. Thus, we hypothesize that the generated circulating IL-1β would promote ER stress- induced myocytes apoptosis via IRAK-2/CHOP pathway. In order to testify our hypothesis, we implemented both in vivo and in vitro experiments. In the in vitro study, ER stress- induced apoptosis was evaluated in primary myocytes which were treated with high glucose and IL-1β. The role of IRAK-2 was evaluated by IRAK-2 siRNA knockdown. In the in vivo study, DCM was induced by streptozotocin (STZ) injection. The circulating IL-1β was detected and its role in ER stress was further investigated by administration of IL-1β antagonist. We believe that results in the current study would be significant in interpretating the association between inflammation and DCM and benefit indentifying potential novel molecular targets for DCM therapy.

## Methods

### Cell culture

Myocytes were isolated form neonate male Sprague–Dawley (SD) supplied by the Experimental Animal Center of Xi’an Jiaotong University. The isolation procedure was in accordance with the description in our previous studies [11]. Briefly, the hearts were harvested after rats were sacrificed. Heart was treated by perfusion with Liberase (4.5 mg/ml, Roche). Cells were cultured in medium containing minimum essential medium (MEM) containing Hank’s buffered salt solution (HyClone), bovine calf serum (5 %, Gibco), l-glutamine (2 mmol/L, Invitorgen), CaCl_2_ (1.8 mmol/L, Sigma), penicillin/streptomycin (100 U/ml, Sigma) and 2,3-butanedione monoxime (10 mmol/L, Sigma) for 1 h. Then the medium was replaced by medium containing MEM (Gibco), myocyte bovine serum albumin (0.1 mg/ml, Invitrogen), l-glutamine (2 mmol/L, Invitrogen) and penicillin/streptomycin (100 U/ml, Sigma) in an incubator proving humidified environment and fresh air (0.5 % CO_2_) at 37 °C.

### Small interfering RNA (siRNA) treatment

In this study, gene of IRAK2 was silenced by using RNA interfering (RNAi) technique by siRNA. Targeting sequence of siRNA against *irak*-*2* was 5′-CTTCGCCTCCTACGTGATCAC-3′ (GenePharma). The scramble siRNA was 5′-GAAACAGACGACGTTGACAA-3′ (GenePharma) which was used as negative control [14]. siRNA was transfected into myocytes at final concentration of 12.5 mmol/L with the assistance of HiPerFect siRNA transfection reagent (Qiagen) according to manufacturer’s instructions. After 24-h incubation, the cells were ready for subsequent experiments.

### Cell treatment

When cell populations reached confluence above 60 %, the cultured cells were respectively incubated with normal glucose medium (D-type glucose concentration at 5.5 mmol/L) or high glucose medium (D-type glucose concentration at 33 mmol/L) for 48 h and/or IL-1β (20 ng/ml, sigma) for 12 h.

### Animals

Thirty 10-week old male SD rats with mean body weight at (305 ± 15) g (Animal Experimental Center of Xi’an Jiaotong University) were used in this study. The mean fasting glucose concentration of the selected animals was (113.29 ± 25.17) mg/dL. Animals were raised in independent polypropylene cages under controlled conditions (artificial alternating 12-h dark- light cycle, temperature at 25 ± 1 °C, humidity at 65 ± 4 %) for 1 week prior to experiments. The animals were free to standard rodent food and sterilized water. All animal experimental procedures were carried out according to protocols approved by Medical Animal Research Ethics Committee at Xi’an Jiaotong University.

### Animal treatment

**Fig. 1:**
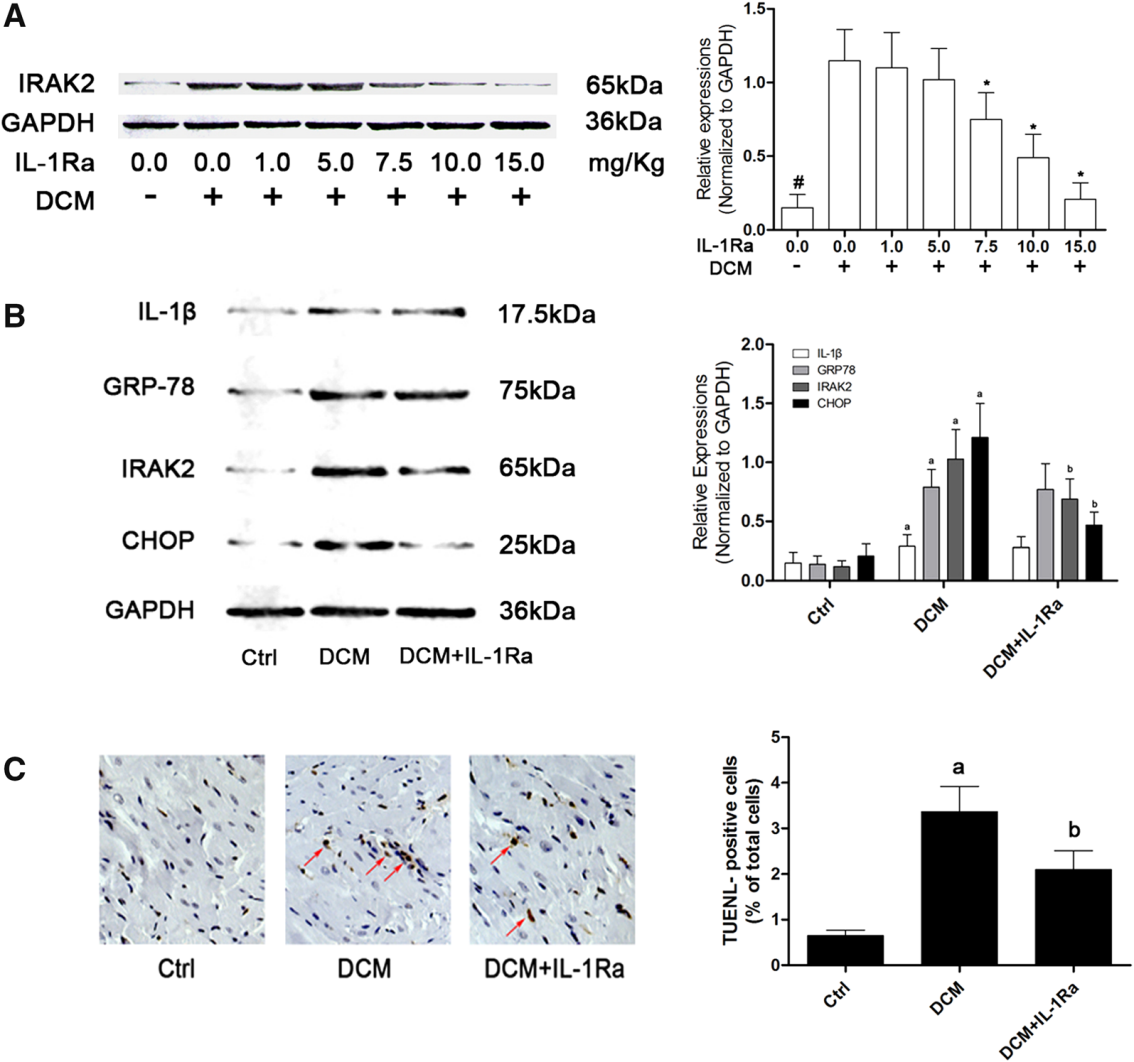
**A** The *right side* of **A** demonstrated immunoblots of IRAK-2 and GAPDH in protein extracted from cardiac tissue from control rats, DCM rats, DCM rats received IL-1Ra administration at dosages of 1.0, 5.0, 7.5, 10.0 and 15.0 mg/Kg respectively daily for 1 week. *Columns* on the *right part* indicated the relative expression levels of IRAK-2 [*hash* differences were significant when compared with control rats (p < 0.05); *asterisk* differences were significant when compared with previous dosages (p < 0.05)]. **B** demonstrated the immunoblots of IL-1β, GRP-78, IRAK-2, CHOP and GAPDH in myocardial tissue in Ctrl, DCM and DCM + IL-1Ra respectively. *Columns* on the *right side* indicated the relative expression levels of IL-1β, GRP-78, IRAK-2 and CHOP (normalized to GAPDH) respectively. **C** Captured images demonstrated TUNEL staining in myocardial tissue harvested from Ctrl, DCM and DCM + IL-1Ra respectively. The *red arrows* are pointing at TUNEL-positive cells. *Columns* on the *right side* indicated the percentage of TUNEL-positive cells in Ctrl, DCM and DCM + IL-1Ra respectively [*letter a* differences were significant compared with Ctrl (p < 0.05); *letter b* differences were significant when compared with DCM (p < 0.05)]

STZ injections (250/20 ml) were acquired from CSPC Pharma. Diabetes of rats was induced by single intraperitoneal injection of STZ (65 mg/Kg bodyweight, dissolved in sodium citrate buffer with pH = 4.5); recombinant IL-1Ra (10 mg/Kg bodyweight, Sigma) was administrated subcutaneously once per day for 1 week according to previous studies [18] and our pre-experiments (Fig. 1A). Equal volume of physiological saline (equaled to the volume of used STZ injection) was tittered into sodium citrate buffer solution and administrated as control. Diabetic animal model establishment was confirmed by fasting blood glucose concentration tests via tail vein (One Touch SureStep Meter, LifeScan) 4 weeks after STZ injections. 6 weeks after modeling, the invasive hemodynamic cardiac function evaluation was carried out and the rats were sacrificed to acquire samples.

### Invasive hemodynamic cardiac function evaluation

After anesthetized by intraperitoneal injection of chloral hydrate (10 %, 0.03 ml/Kg bodyweight), invasive hemodynamic determination was conducted in accordance with our previous studies [11]. Through the right carotid artery, a Mikro Tip catheter transducer (Millar Instruments) was intubated into left ventricle and connected to Powerlab 4/25 Biological Analysis System (AD Instrument), then the left ventricular systolic pressure (LVSP) and left ventricular end- diastolic pressure (LVEDP) were measured and recorded.

### IL-1β concentration determination

IL-1β concentration in serum or cell culture supernatants were determined by using specific rat enzyme linked-immuno-sorbent assay (ELISA) kit “Quantkine Rat IL-1β” (R&D). The detection procedure was carried out according to the manufacturer’s instructions. The concentration results were calculated based on the absorbance value and standard curves.

### Cardiomyocyte apoptosis assessment

#### Hoechst staining

In cultured myocytes, Hoechst fluorescent staining was used to detect the apoptosis. Cultured cells were harvested and fixed by paraformaldehyde (4 %) at room temperature for 1 h. Then cTnI (Abcam) conjugated with Alexa Fluor 488 (Abcam) and Hoechst 33342 (5 μmol/L, Sigma) was used to incubate the myocytes at 37 °C for 30 min in a humidified dark chamber. Then the fluorescent images were captured by a fluorescence microscope (Nikon).

#### TUNEL staining

Myocytes apoptosis in cardiac tissue was examined by terminal dUTP trasferase nick end labeling (TUNEL). The paraffin- embedded 5-μm thick sections of cardiac tissue were digested by proteinase K (20 μmol/L, Sigma) and fixed by paraformaldehyde (4 %, Solarbio) and then permeabilized by Triton X-100 (0.1 %, Sigma- Aldrich). A TUNEL assay kit (Roche) was employed to detect the apoptotic cells per manufacturer’s instructions. Briefly, the cryo- sections were washed by PBS and then incubated with prepared TUNEL solution for 1 h at 37 °C in a dark chamber. The TUNEL-positive cells were observed and counted under a light microscope (Motic). For in vitro apoptosis evaluation, the In Situ Cell Death Detection kit (Roche) was used. Briefly, the cultured myocytes were fixed with paraformaldehyde (4 %, Solarbio) and then permeabilized with Triton X-100 (0.1 %, Sigma- Aldrich). According to the manufacturer’s protocols, the cells were incubated with TUENL staining solution for 1 h. The nuclei were stained with DAPI (Beyotime). The TUNEL positive cells were observed and counted under a fluorescence microscope (Nikon).

### Western blotting

The homogenates were acquired after cardiac tissue or myocytes were homogenized by RIPA lysis buffer system (Santa Cruz) with PMSF (Santa Cruz). After centrifugation, the supernatants were separated from the homogenates. Protein concentration in supernatants was measured by a BCA protein assay kit (Pierce). The protein was separated by vertical electrophoresis in SDS gels and then transferred to polyvinylidene fluoride (PVDF) membranes. Specific antibodies against GRP-78 (Abcam), CHOP (Cell Signaling Tech), IRAK-2 (Cell Signaling Tech), IL-1β (Abcam) and GAPDH (Santa Cruz) were used to incubate the membranes at 4 °C for 12 h. After washed, second antibody conjugated to HRP (Santa Cruz) was used to incubate the membranes at room temperature for 2 h. Eventually, after developed by Super Signal West Pico chemiluminsecence reagent (Thermo Scientific), the immunoblots were visualized on X-ray films. ImageJ2x software was used to quantify the intensities of the immunoblots.

### Statistical analysis

Data acquired in this study was presented in a (mean ± SD) manner and further analyzed by software SPSS (version 17.0, SPSS). Differences between groups were analyzed by one- way analysis of variance (ANOVA) followed by a LSD test. P < 0.05 was considered statistically significant.

## Results

### IL-1β level in myocytes was not elevated by high- glucose incubation

**Fig. 2:**
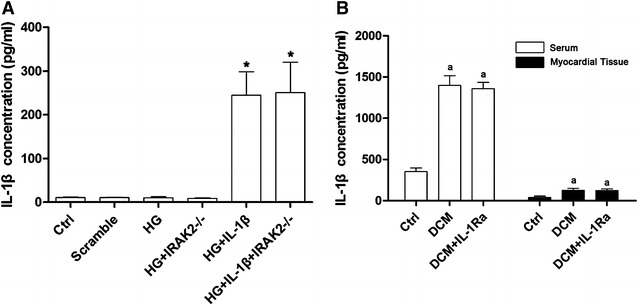
**A** *Columns* in this figure demonstrated the IL-1β level in supernatant of cultured primary myocytes by ELISA. According to different treatments, equal amount myocytes were divided into Ctrl (control), Scramble (cells transfected with scramble siRNA), HG (cells incubated by medium with glucose concentration at 33 mmol/L), HG + IRAK2^−/−^ (cells treated with high-glucose medium and transfected with siRNA targeting *irak2*), HG + IL-1β (cells treated with high-glucose medium and exogenous IL-1β) and HG + IL-1β + IRAK2^−/−^ (cells treated with high-glucose medium and exogenous IL-1β and transfected with siRNA against *irak2*) respectively [*asterisk* differences were significant when compared with HG (p < 0.05]. **B** *Columns* in this part showed the detected IL-1β in serum and myocardial tissue supernatant collected from normal control (Ctrl), DCM rats (DCM) and DCM rats received IL-1Ra administration (DCM + IL-1Ra) respectively [*letter a* differences were significant when compared with Ctrl (p < 0.05)]

As shown in Fig. 2A, after high-glucose incubation, the IL-1β level in myocytes was not elevated significantly. Suggesting the myocytes were not the source of IL-1β production under circumstances of DCM.

High- glucose mediated ER stress- induced apoptosis was elevated by IL-1β which was inhibited by IRAK-2 silencing.

**Fig. 3:**
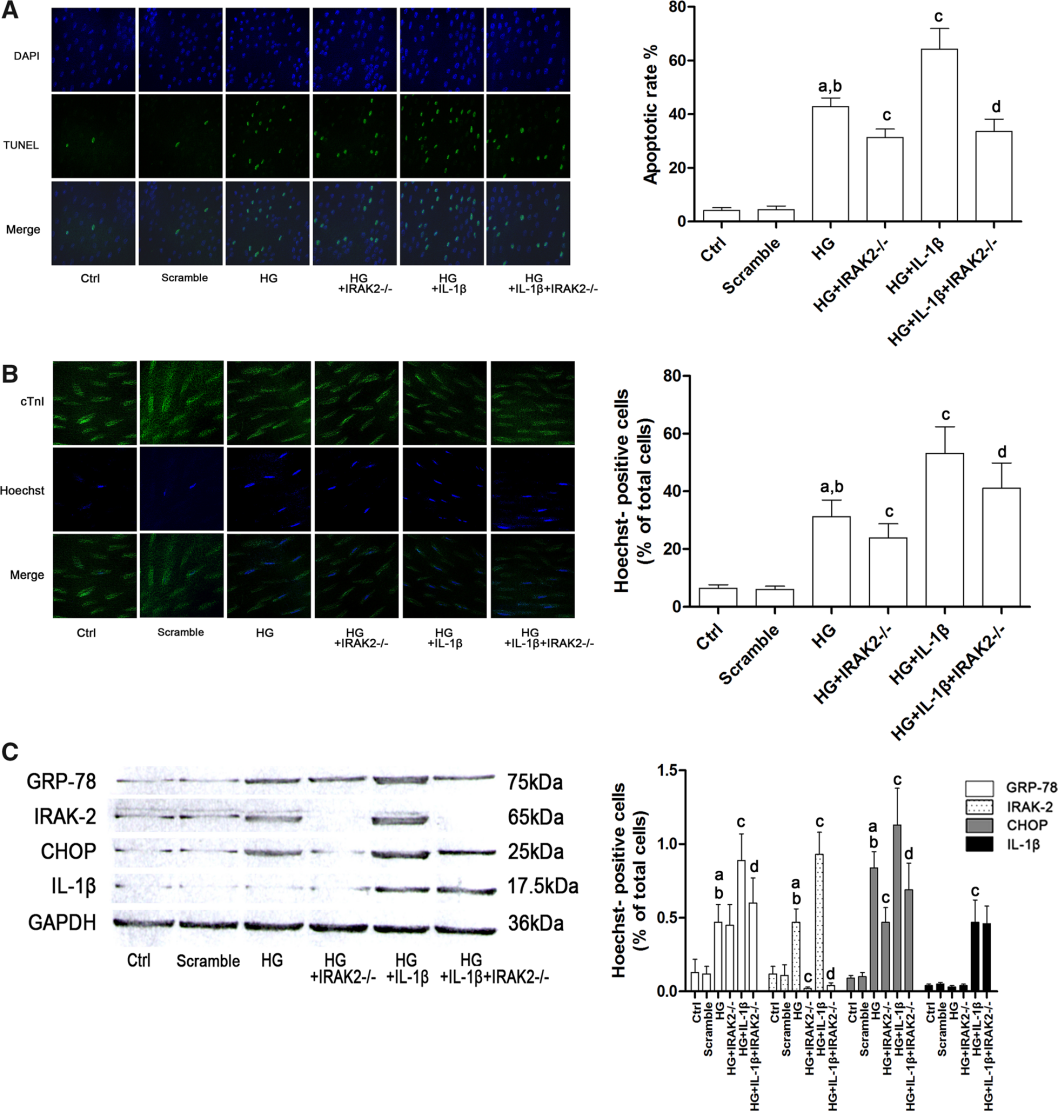
**A** Demonstrated the captured images of DAPI, TUNEL and their merged images in cultured myocytes. DAPI staining indicated the location of cell nuclei; TUNEL positive cells indicated the apoptotic cells. *Column* in the *left part* of **a** indicated the calculated apoptotic rate of myocytes in Ctrl, Scramble, HG, HG + IRAK2^−/−^, HG + IL-1β and HG + IL-1β + IRAK2^−/−^ respectively. **B** Demonstrated the captured images of cTnI (conjugated with Flour 488), Hoechst and their merged images in cultured myocytes. cTnI positive cells were identified as myocytes; Hoechst positive ones were identified as apoptotic cells. Columns in on the right part stood for percentage of Hoechst-positive cells in Ctrl, Scramble, HG, HG + IRAK2^−/−^, HG + IL-1β and HG + IL-1β + IRAK2^−/−^ respectively. **C** demonstrated the immunoblots of GRP-78, IRAK-2, CHOP, IL-1β and GAPDH respectively in total protein extracted from myocytes. Columns on the right part of the figure demonstrated the relative expression levels (normalized to GAPDH) of GRP-78, IRAK-2, CHOP and IL-1β in Ctrl, Scramble, HG, HG + IRAK2^−/−^, HG + IL-1β and HG + IL-1β + IRAK2^−/−^ respectively [*letter a* differences were significant when compared with Ctrl (p < 0.05); *letter b* differences were significant compared with Scramble; *letter c* differences were significant compared with HG (p < 0.05); *letter d* differences were significant compared with HG + IL-1β (p < 0.05)]

The apoptosis of isolated myocytes was evaluated by both Hoechst and TUNEL staining. The results of TUNEL staining were demonstrated in Fig. 3A while the results of Hoechst staining were demonstrated in Fig. 3B. After high- glucose incubation, GRP-78 and CHOP expression levels as well as the apoptotic rate increased significantly. Extra IL-1β further boosted GRP-78 and CHOP expression levels as well as the apoptotic rate. However, the IRAK-2 silenced myocytes showed resistance to IL-1β and/or high- glucose incubation- induced CHOP expression elevation and subsequent apoptosis without affecting the expression level of GRP-78.

IL-1β expression level was found elevated in both circulation and cardiac tissue in DCM rats.

**Fig. 4:**
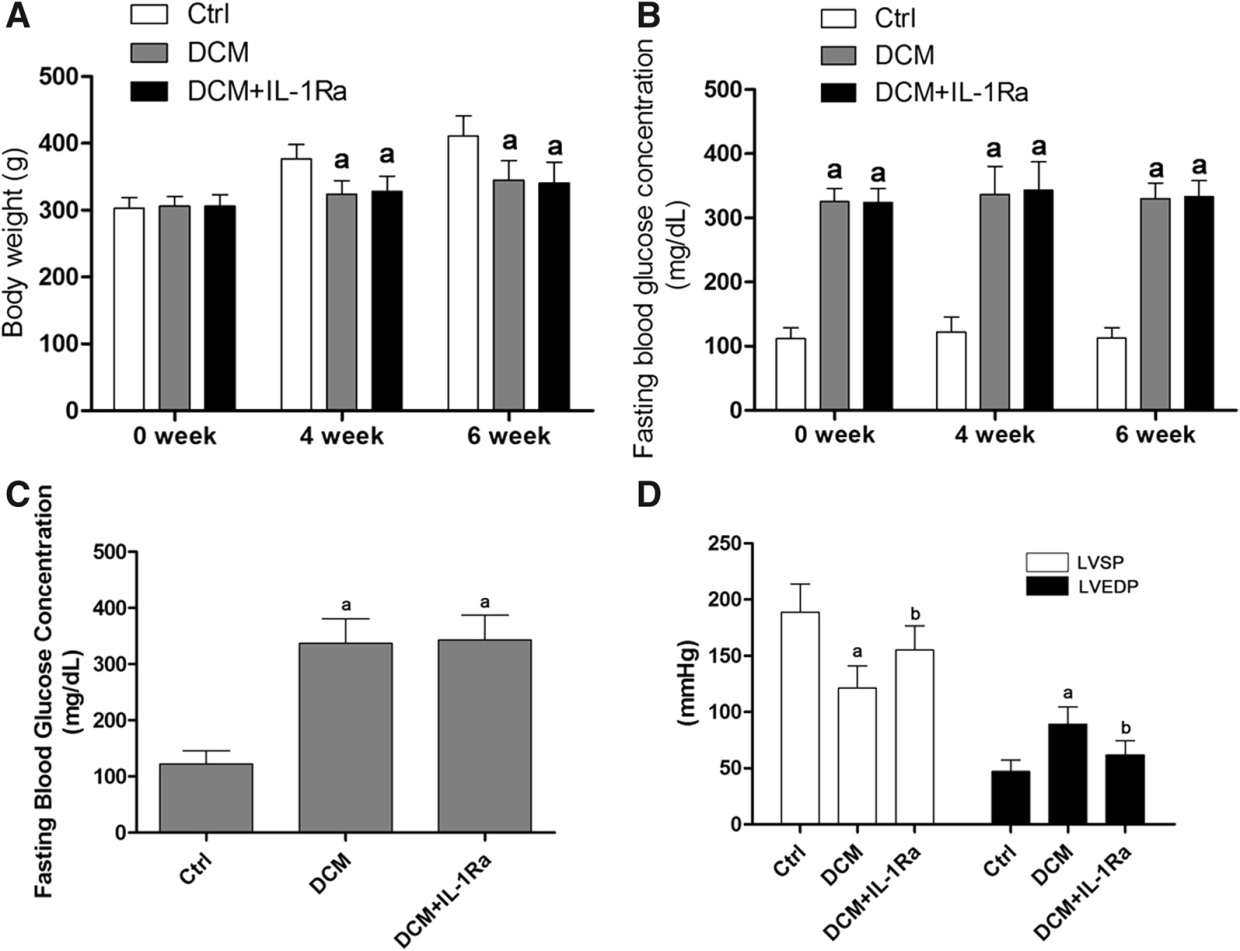
**A** *Columns* indicated the bodyweight of rats in Ctrl (control), DCM (DCM rat model) and DCM + IL-1Ra (DCM rats received IL-1Ra treatment) measured at 0, 4 and 6 weeks after modeling. **B** *Columns* indicated the fasting blood glucose concentrations of rats in Ctrl, DCM and DCM + IL-1Ra measured at 0 week, 4 weeks and 6 weeks after modeling. **C** Columns stood for the fasting blood glucose concentrations in Ctrl, DCM and DCM + IL1Ra measured at 4 weeks after modeling respectively. **D** columns in this figure demonstrated detected LVSP (*white columns*) and LVEDP (*black columns*) by hemodynamic examinations in Ctrl, DCM and DCM + IL-1Ra measure at 6 weeks after modeling respectively [*letter a* differences were significantly compared with Ctrl (p < 0.05); *letter b* differences were significant compared with DCM (p < 0.05)]

The establishment of DCM rat model was verified by fasting blood glucose concentration and impaired cardiac function (Fig. 4D). As demonstrated in Fig. 2B, evidenced by results from ELISA, the levels of IL-1β was found dramatically increased in both serum and cardiac tissue in DCM rats.

The IL-1R antagonist, IL-1Ra, significantly improved cardiac function in DCM.

As shown in Fig. 4C, the average fasting blood glucose concentration of animal received STZ administration was higher than 300 mg/dL which was deemed to be diabetic in rodents [19, 20]. The cardiac function of DCM was also impaired evidenced by elevated LVEDP and reduced LVSP (Fig. 4D). However, in DCM rats received IL-1Ra administration, the cardiac function was improved significantly even with uncontrolled high- fasting glucose concentration (Fig. 4D).

IL-1Ra lowered IRAK2/CHOP induced apoptosis in DCM heart without affecting circulating and myocardial IL-1β levels.

This result was demonstrated in Fig. 1. Compared with DCM rats, there were not significant differences of expression levels of cardiac GRP78 (Fig. 1B), IL-1β (Fig. 2B) and serum IL-1β (Fig. 2B) in DCM rats received IL-1Ra administration. However, in cardiac tissue of DCM rats administrated with IL-1Ra, the expression levels of CHOP and IRAK2 decreased significantly. As a result, the cardiac apoptosis of IL-1Ra treated DCM rats was dramatically decreased compared with DCM rats.

## Discussion

The situation of preventing and treating diabetes is grave not only because of the sustained increasing of morbidity of diabetes, but also due to the high mortality resulted from various complications of diabetes [21]. It was estimated in the report from the World Health Organization (WHO) that there would be over 300 million diabetic patients by 2025 [22]. Among the diabetic complications, cardiovascular complications were considered the major causes responsible for the mortality of diabetes. Independent from other cardiovascular diseases including congenital heart disease, coronary artery disease, hypertensive heart disease, valvular heart disease and other secondary heart diseases, DCM is characterized by cardiac function impairment and termed as one of the clinical manifestations of diabetes [23].

Uncontrolled and sustained hyperglycemia is the pathological basis of both type-1 and type-2 diabetes. It has been established that excessive blood glucose impaired cardiac function via various mechanisms, of which ER stress took indispensable roles in inducing myocytes apoptosis, resulting in contractile unit loss [24]. As one of the characterized cardiovascular complications of diabetes, DCM was found closely associated with ER stress [25]. Recently, ER stress and inflammation have been linked to various human diseases because effectors and regulators are extensively shared by ER stress and inflammatory signaling pathways which were found to exacerbate cell apoptosis and dysfunctions in a “vicious cycle” [26]. More and more evidences suggest that islet β-cell dysfunction and loss would induce oxidative stress, glucotoxicity, lipotoxicity, mitochondrial dysfunction and inflammation [27–30]. It is hard to decide which of the mechanisms is the most dominant, but inflammation is confirmed closely associated with DCM [31]. Thus, it is reasonable to believe that DCM belongs to the inflammatory diseases.

Among the pro-inflammatory cytokines, IL-1 was found particularly important to diabetes because most elevated circulating pro-inflammatory factors in diabetic patients were considered IL-1 dependent, and IL-1 blocking was reported to reduce their circulating concentrations [32]. Generally, IL-1 is suggested as the product of immunocytes such as T cells, but β-cell produced IL-1β was also observed in pancreatic secretions from patients with diabetes [33]. It was also found that hyperglycemia yielded the IL-1β production from human islet β-cells, resulting in decreased β-cell proliferation, increased β-cell dysfunction and apoptosis [34]. Bone marrow- derived macrophages was reported also contributed in IL-1β production in diabetes [35]. In this study, in accordance with the above mentioned studies, we also observed the elevation of circulating IL-1β in DCM rats. However, in high- glucose incubated myocytes, IL-1β level was found unchanged. This result indicated that myocytes was not the source of IL-1β in DCM. Furthermore, myocardial IL-1β level was elevated in DCM heart, suggesting excessively produced IL-1β affected heart via circulation.

Applications of antagonists of IL-1β demonstrated significant therapeutic effects on diabetic cardiovascular complications both in experimental animals [36] and human [37] indicating that IL-1β played an important role in initiating and exacerbating cardiovascular complications of diabetes. IL-1β activates its down-stream signaling by binding to its receptor- IL-1R. Then the activated receptor binds to the accessory protein, AcP, to form IL-1R-AcP complex. The C- terminal death- domain then couples and activates IRAKs to initiate tumor necrosis factor receptor associated factor-6 (TRAF-6) [38], leading to activate down- stream apoptotic pathway [39]. Interestingly, a recent study identified the correlation between ER stress- induced apoptosis and IRAK-2. On one hand, the expression level of IRAK-2 was elevated under condition of ER stress, on the other hand, increased IRAK-2 boosted the expression of CHOP, leading to cell apoptosis [14]. Rather than other two transducers, namely protein kinase RNA- like ER kinase (PERK) and activating transcription factor-6 (ATF-6), inositol requiring enzyme-1 (IRE1) was supposed to be the signaling linking ER stress and IRAK-2 [14].

In the in vitro part, the IL-1β incubation exacerbated the high- glucose induced myocytes apoptosis though increase the expression of IRAK-2 which in turn further increased cell death by elevating CHOP expression. In *irak2* silenced myocytes, however, the aggravating effect of IL-1β on ER stress- induced apoptosis was impaired. These results indicated that IL-1β promoted ER stress-induced myocytes apoptosis in DCM via IRAK-2. In the in vivo part, after DCM rats were administrated with the IL-1Ra, both of CHOP expression and apoptosis were alleviated even the hyperglycemia was still sustained and the ER stress was not relieved. As a result, the cardiac function of DCM rats was significantly improved.

In summary, we could draw several conclusions according to the results in the current study. Firstly, cardiac function was impaired by elevated circulating IL-1β which induced myocytes death. Secondly, in DCM, apoptotic signals were transduced through IL-1β/IRAK2/CHOP pathway. This pathway could be considered as a crosstalk between IL-1 and ER stress signaling. Finally, suppressing IL-1β production or signaling could be considered as potential molecular targets- based therapeutic strategies for DCM.

### Limitations and prospectives

There are also limitations for the present study. Firstly, in this study, in order to investigate the association between high glucose environment and pathological mechanisms of myocytes apoptosis, STZ injection was used to establish the animal model of diabetes by inducing islet cell lose. However, by this method, instead of type-2 diabetes which is the most common type of diabetes is type-2 diabetes in human, type-1 diabetes was modeled. Though the mechanisms would be much more complex in DCM of type-2 diabetes because other factors such as oxidative stress and lipid metabolism disorder are involved, further investigation should be implemented. Secondly, it was not adequate enough to guarantee the solidity of the conclusions due to the unavailability of *irak2* gene knock-out animals. We are planning to investigate the molecular mechanisms of DCM concerning inflammation and myocyte apoptosis in type-2 diabetes animal model in the very near future. More studies would be carried out as soon as we acquire the *irak2* knock-out animals.

## Conclusions

Results from this study suggest that elevated circulating inflammatory cytokine such as IL-1β contributed to the cardiac contractile unit loss in diabetic heart, which takes responsibility for impairment of cardiac function. Further investigation indicates that IL-1β promoted endoplasmic reticulum stress- induced myocytes apoptosis through IL-1β/IRAK2/CHOP pathway in DCM. These results would not only deepen our understanding of mechanisms of DCM, but also provide new clues for choosing therapeutic molecular target in the future.

## References

[1] Boudina S, Abel ED (2007). Diabetic cardiomyopathy revisited. Circulation.

[2] Bendas A, Rothe U, Kiess W, Kapellen TM, Stange T, Manuwald U, Salzsieder E, Holl RW, Schoffer O, Stahl-Pehe A (2015). Trends in incidence rates during 1999–2008 and prevalence in 2008 of childhood type 1 diabetes mellitus in GERMANY—model-based national estimates. PLoS One.

[3] Cai L, Kang YJ (2003). Cell death and diabetic cardiomyopathy. Cardiovasc Toxicol.

[4] Poirier P, Bogaty P, Philippon F, Garneau C, Fortin C, Dumesnil JG (2003). Preclinical diabetic cardiomyopathy: relation of left ventricular diastolic dysfunction to cardiac autonomic neuropathy in men with uncomplicated well-controlled type 2 diabetes. Metabolism.

[5] Liu ZW, Zhu HT, Chen KL, Dong X, Wei J, Qiu C, Xue JH (2013). Protein kinase RNA-like endoplasmic reticulum kinase (PERK) signaling pathway plays a major role in reactive oxygen species (ROS)-mediated endoplasmic reticulum stress-induced apoptosis in diabetic cardiomyopathy. Cardiovasc Diabetol.

[6] Donath MY, Shoelson SE (2011). Type 2 diabetes as an inflammatory disease. Nat Rev Immunol.

[7] Banerjee M, Saxena M (2012). Interleukin-1 (IL-1) family of cytokines: role in type 2 diabetes. Clin Chim Acta.

[8] Rhodes CJ (2005). Type 2 diabetes-a matter of beta-cell life and death?. Science.

[9] Shen Y, Qin J, Bu P (2015). Pathways involved in interleukin-1beta-mediated murine cardiomyocyte apoptosis. Tex Heart Inst J.

[10] Harding HP, Ron D (2002). Endoplasmic reticulum stress and the development of diabetes: a review. Diabetes.

[11] Liu Z, Cai H, Zhu H, Toque H, Zhao N, Qiu C, Guan G, Dang Y, Wang J (2014). Protein kinase RNA-like endoplasmic reticulum kinase (PERK)/calcineurin signaling is a novel pathway regulating intracellular calcium accumulation which might be involved in ventricular arrhythmias in diabetic cardiomyopathy. Cell Signal.

[12] Xu J, Zhou Q, Xu W, Cai L (2012). Endoplasmic reticulum stress and diabetic cardiomyopathy. Exp Diabetes Res.

[13] Li Y, Guo Y, Tang J, Jiang J, Chen Z (2014). New insights into the roles of CHOP-induced apoptosis in ER stress. Acta Biochim Biophys Sin.

[14] Benosman S, Ravanan P, Correa RG, Hou YC, Yu M, Gulen MF, Li X, Thomas J, Cuddy M, Matsuzawa Y et al: Interleukin-1 receptor-associated kinase-2 (IRAK2) is a critical mediator of endoplasmic reticulum (ER) stress signaling. PLoS One 2013; 8(5). 10.1371/journal.pone.0064256 PMC3665826 23724040

[15] Li YL, Guo FK, Wu SG (2000). Effects of antisense IRAK-2 oligonucleotides on PGI2 release induced by IL-1 and TNF. Acta Pharmacol Sin.

[16] Dayer JM (2002). Evidence for the biological modulation of IL-1 activity: the role of IL-1Ra. Clin Exp Rheumatol.

[17] Deguine J, Barton GM (2014). MyD88: a central player in innate immune signaling. F1000Prime Rep.

[18] Xu C, Shen J, Zhang J, Jia Z, He Z, Zhuang X, Xu T, Shi Y, Zhu S, Wu M (2015). Recombinant interleukin-1 receptor antagonist attenuates the severity of chronic pancreatitis induced by TNBS in rats. Biochem Pharmacol.

[19] Li Y, Zhang Y, Liu DB, Liu HY, Hou WG, Dong YS (2013). Curcumin attenuates diabetic neuropathic pain by downregulating TNF-alpha in a rat model. Int J Med Sci.

[20] Wu J, Yan LJ (2015). Streptozotocin-induced type 1 diabetes in rodents as a model for studying mitochondrial mechanisms of diabetic beta cell glucotoxicity. Diabetes Metab Syndr Obes.

[21] Xu RS (2015). Pathogenesis of diabetic cerebral vascular disease complication. World J Diabetes.

[22] King H, Aubert RE, Herman WH (1998). Global burden of diabetes, 1995–2025: prevalence, numerical estimates, and projections. Diabetes Care.

[23] Lam CS. Diabetic cardiomyopathy: an expression of stage B heart failure with preserved ejection fraction. Diab Vasc Dis Res. 2015 (pii: 1479164115579006). 10.1177/1479164115579006 25908570

[24] Lakshmanan AP, Harima M, Suzuki K, Soetikno V, Nagata M, Nakamura T, Takahashi T, Sone H, Kawachi H, Watanabe K (2013). The hyperglycemia stimulated myocardial endoplasmic reticulum (ER) stress contributes to diabetic cardiomyopathy in the transgenic non-obese type 2 diabetic rats: a differential role of unfolded protein response (UPR) signaling proteins. Int J Biochem Cell Biol.

[25] Guo R, Liu W, Liu B, Zhang B, Li W, Xu Y (2015). SIRT1 suppresses cardiomyocyte apoptosis in diabetic cardiomyopathy: an insight into endoplasmic reticulum stress response mechanism. Int J Cardiol.

[26] Cao SS, Luo KL, Shi L (2015). Endoplasmic reticulum stress Interacts with inflammation in human diseases. J Cell Physiol.

[27] Maritim AC, Sanders RA, Watkins JB (2003). Diabetes, oxidative stress, and antioxidants: a review. J Biochem Mol Toxicol.

[28] Park SH, Park JH, Shim HM, Na AY, Bae KC, Lim JG, Song DK (2015). Protection of pancreatic beta-cells against glucotoxicity by short-term treatment with GLP-1. Biochem Biophys Res Commun.

[29] Janikiewicz J, Hanzelka K, Kozinski K, Kolczynska K, Dobrzyn A (2015). Islet beta-cell failure in type 2 diabetes—within the network of toxic lipids. Biochem Biophys Res Commun.

[30] Sivitz WI, Yorek MA (2010). Mitochondrial dysfunction in diabetes: from molecular mechanisms to functional significance and therapeutic opportunities. Antioxid Redox Signal.

[31] Luo B, Li B, Wang W, Liu X, Xia Y, Zhang C, Zhang Y, Zhang M, An F (2014). Rosuvastatin alleviates diabetic cardiomyopathy by inhibiting NLRP3 inflammasome and MAPK pathways in a type 2 diabetes rat model. Cardiovasc Drugs Ther.

[32] Malozowski S, Sahlroot JT (2007). Interleukin-1-receptor antagonist in type 2 diabetes mellitus. N Engl J Med..

[33] Poitout V, Robertson RP (2002). Minireview: secondary beta-cell failure in type 2 diabetes–a convergence of glucotoxicity and lipotoxicity. Endocrinology.

[34] Costes S, Langen R, Gurlo T, Matveyenko AV, Butler PC (2013). beta-Cell failure in type 2 diabetes: a case of asking too much of too few?. Diabetes.

[35] Masters SL, Dunne A, Subramanian SL, Hull RL, Tannahill GM, Sharp FA, Becker C, Franchi L, Yoshihara E, Chen Z (2010). Activation of the NLRP3 inflammasome by islet amyloid polypeptide provides a mechanism for enhanced IL-1beta in type 2 diabetes. Nat Immunol.

[36] Vallejo S, Palacios E, Romacho T, Villalobos L, Peiro C, Sanchez-Ferrer CF (2014). The interleukin-1 receptor antagonist anakinra improves endothelial dysfunction in streptozotocin-induced diabetic rats. Cardiovasc Diabetol.

[37] Howard C, Noe A, Skerjanec A, Holzhauer B, Wernsing M, Ligueros-Saylan M, Thuren T (2014). Safety and tolerability of canakinumab, an IL-1beta inhibitor, in type 2 diabetes mellitus patients: a pooled analysis of three randomised double-blind studies. Cardiovasc Diabetol.

[38] Cenni V, Sirri A, De Pol A, Maraldi NM, Marmiroli S (2003). Interleukin-1-receptor-associated kinase 2 (IRAK2)-mediated interleukin-1-dependent nuclear factor kappaB transactivation in Saos2 cells requires the Akt/protein kinase B kinase. Biochem J.

[39] Meng Q, Zheng M, Liu H, Song C, Zhang W, Yan J, Qin L, Liu X (2012). TRAF6 regulates proliferation, apoptosis, and invasion of osteosarcoma cell. Mol Cell Biochem.

